# Supplementary material for: Comprehensive nutrient analysis in agricultural organic amendments through non-destructive assays using machine learning
Source: PLoS One. 2020 Dec 10;15(12):e0242821. doi: 10.1371/journal.pone.0242821 (PMC7728284; doi:10.1371/journal.pone.0242821)
Supplement: S1 File — (DOCX) [file pone.0242821.s001.docx]

# Supporting information


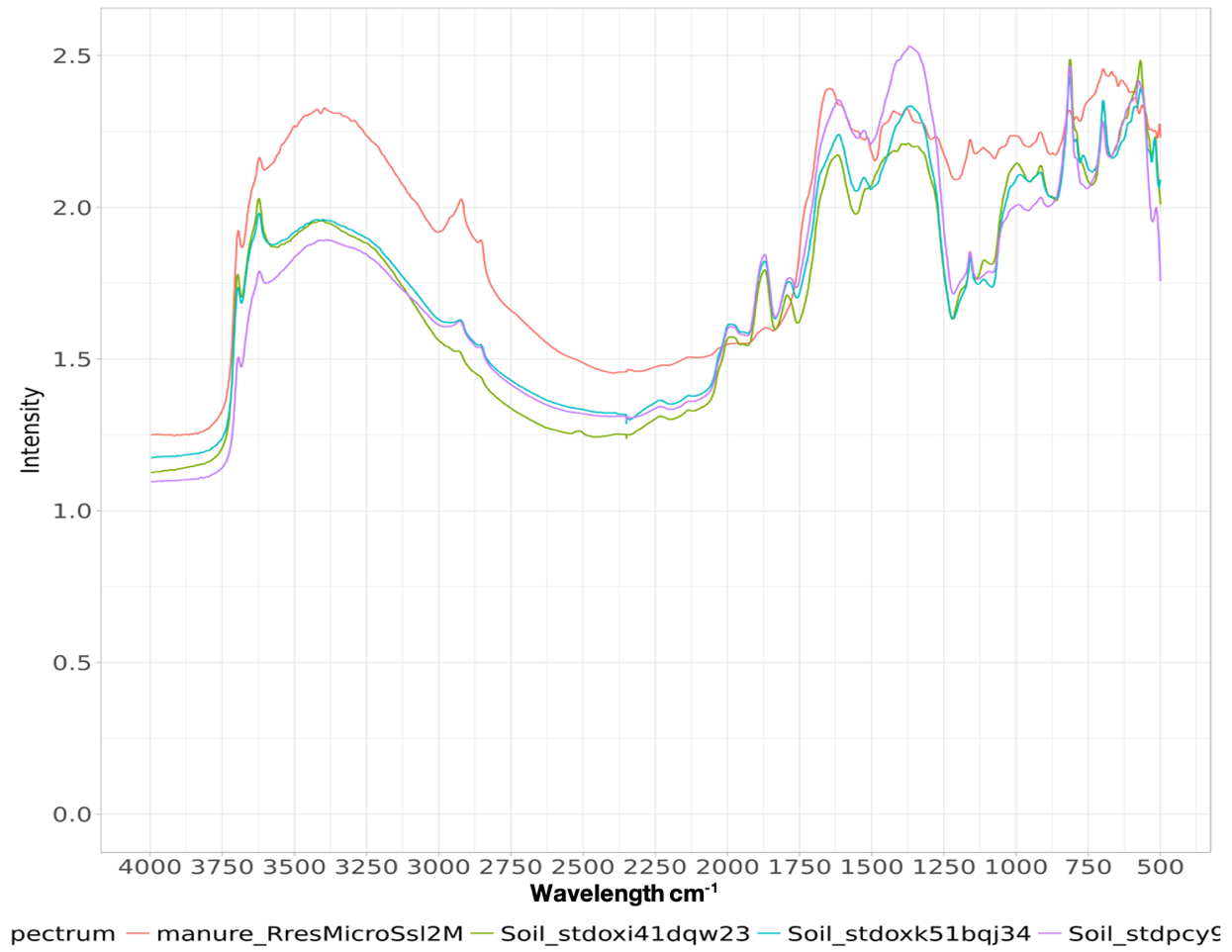


**Wavelength cm^‑1^**

S1 Fig: Organic amendment (OA) sample (in red) with the high ash content (94.9%) has some resemblance to soil MIR spectra of three different soil standards, because there is often some soil mixed in with the OA and some soil features are therefore evident (e.g., O-H stretching in clays at 3694 cm^‑1^).


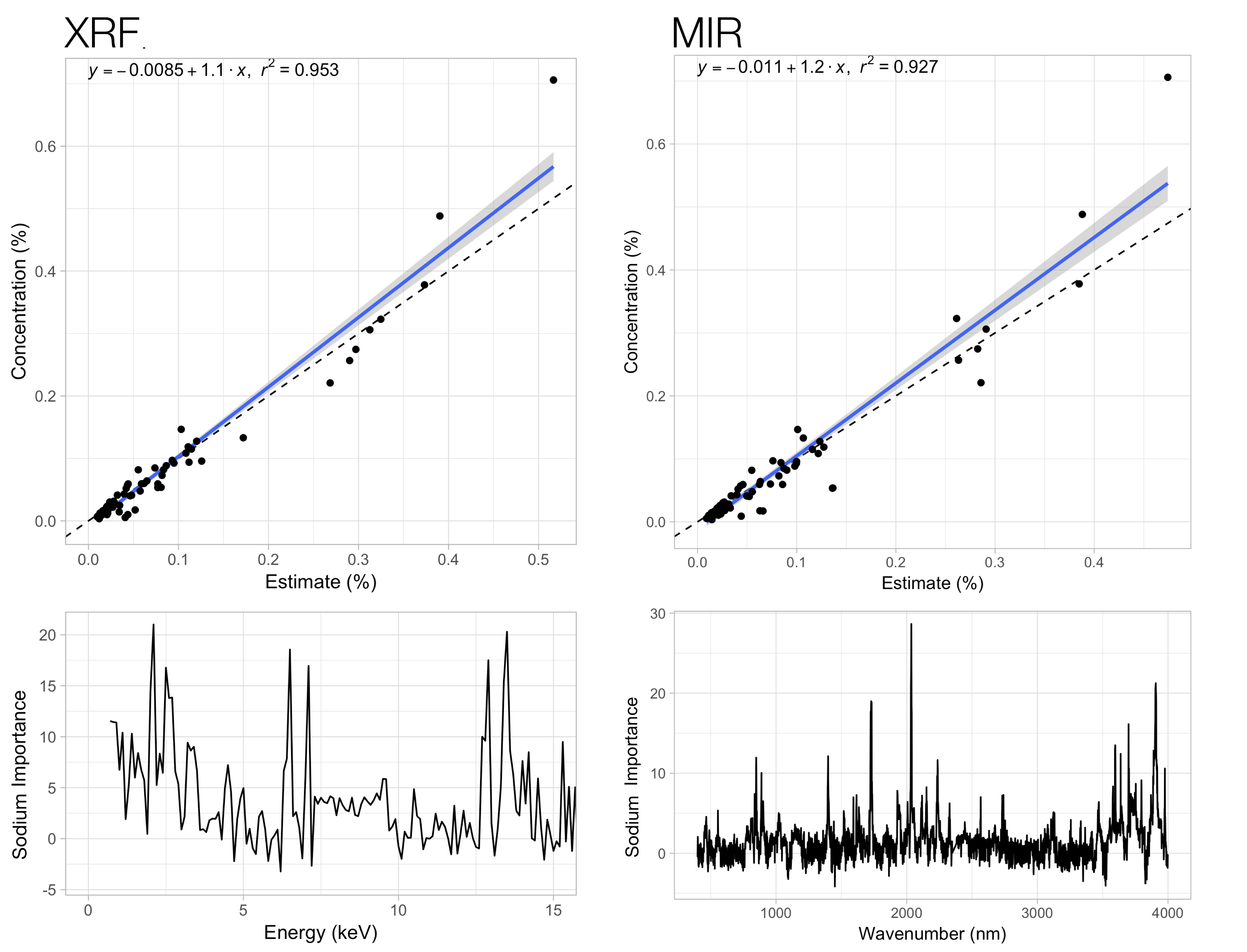


S2 Fig: Whole-spectrum forest regressions for XRF and DRIFT-MIR for sodium in manure on instrument 4473. The dotted line indicates the expected 1:1 ratio for estimates and known values.


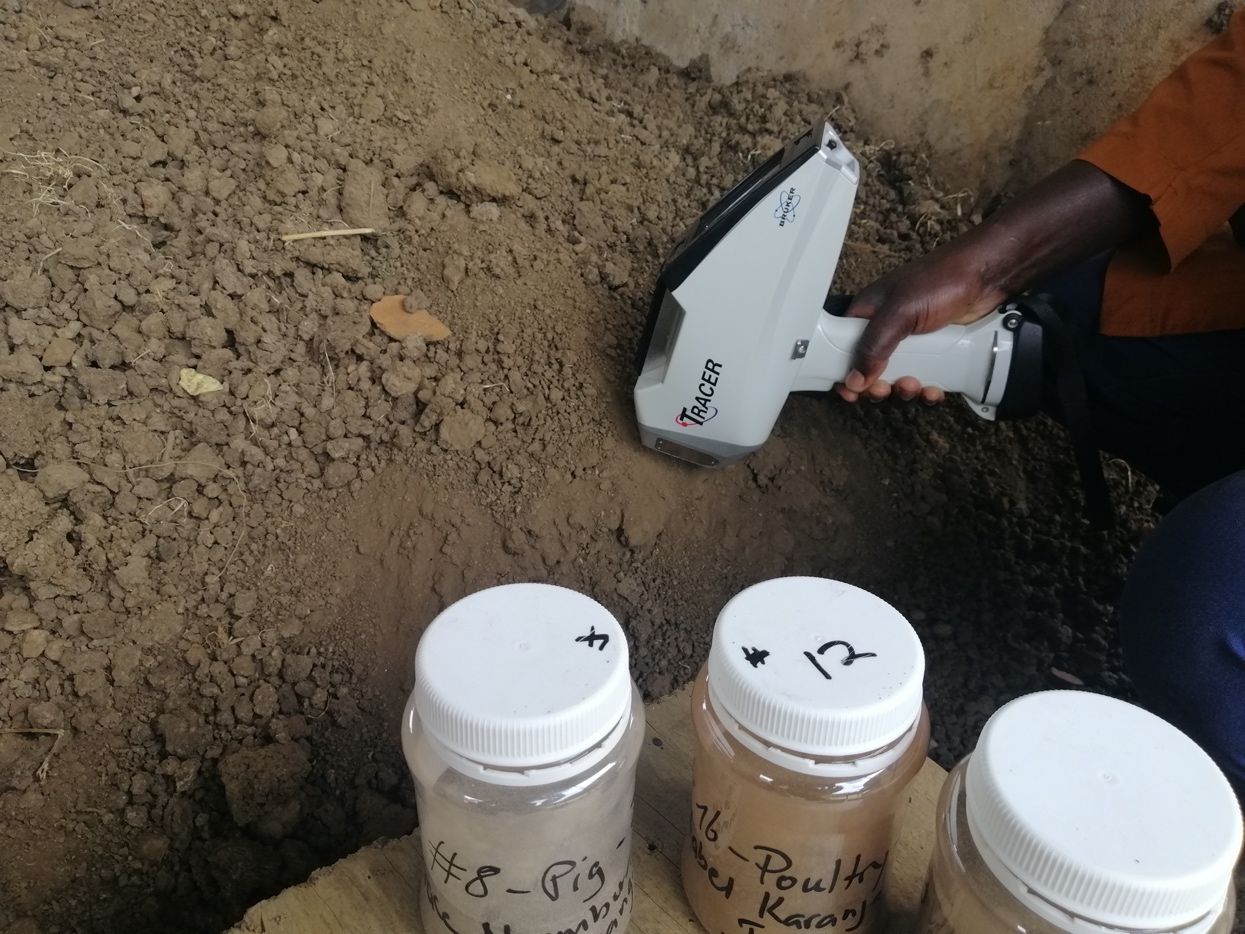


S1 Photo: Tracer 5i pXRF analyzer in live action nature is ideal for screening organic amendments (OA) for nutrients and heavy metals. The Tracer 5i is configured for measuring elements in powdered manure samples which require preparation, and these are best analyzed in a sample cup. It is important to keep in mind that calibrations work best for similar materials with a similar composition to the reference materials used to create the calibration. Measuring conditions and sample preparation should also be the same.


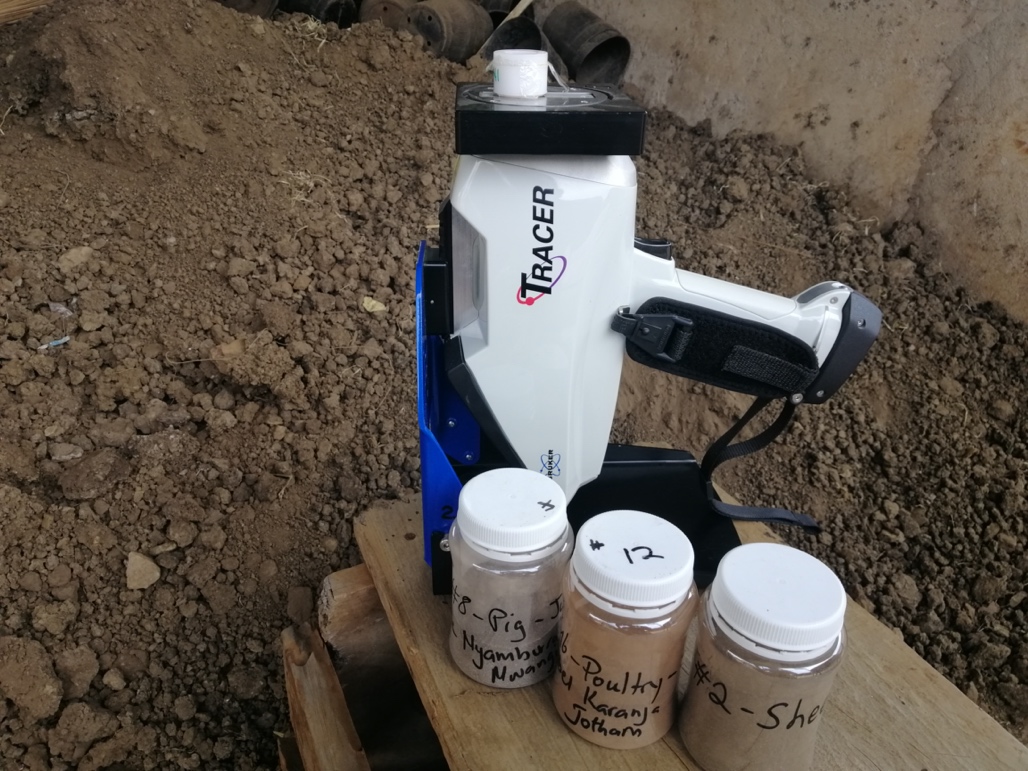


S2 Photo: The Tracer 5i portable XRF (pXRF) analyzers can be configured with calibrations that enable “point-and-shoot” analysis on a variety of OA samples. The present calibrations are meant for multi-elemental analysis of OA samples dried, powdered and packed in a sample cup with 4μm Prolene™ film.


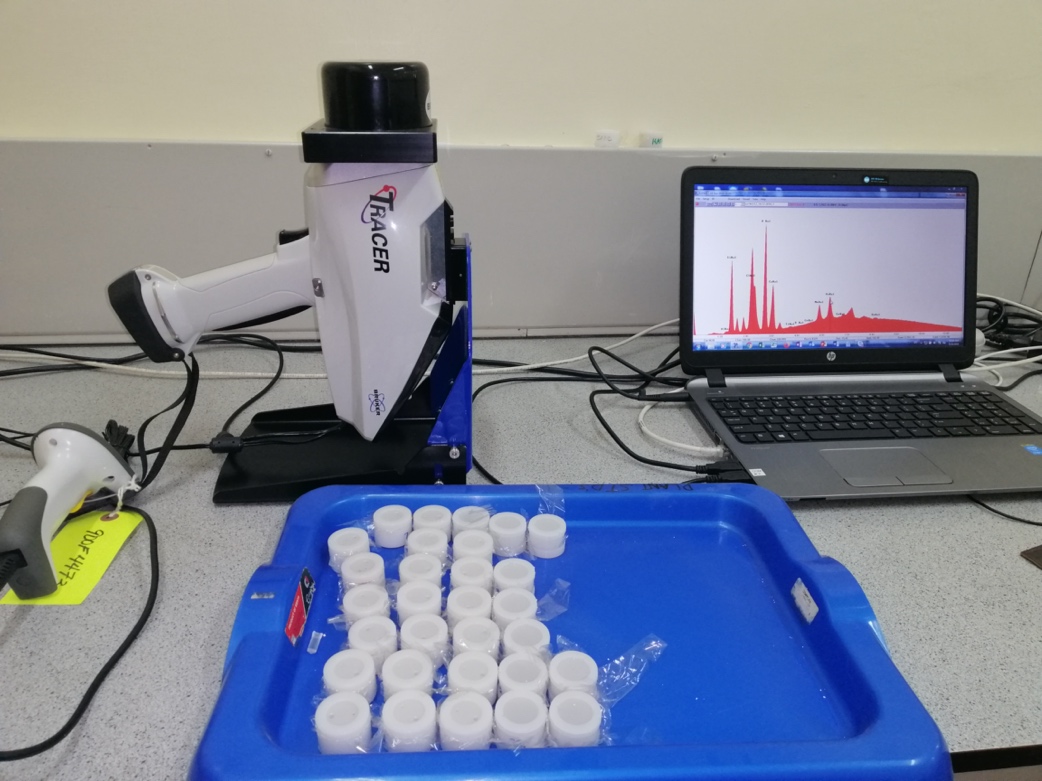


S3 Photo: The Tracer 5i portable XRF (pXRF) analyzer in a lab setup. The most convenient way to use the pXRF for samples analysis is in its desktop configuration. Samples as small as 4g are typically placed on the X-ray film (prolene) wrap of sample cups. Prior to running the test, the analyzer head and sample are covered.
